# Werther effect in the juvenile population. About a series of cases

**DOI:** 10.1192/j.eurpsy.2023.2366

**Published:** 2023-07-19

**Authors:** M. Valverde Barea, A. Alvarado Dafonte, L. Soldado Rodriguez, A. España Osuna

**Affiliations:** 1 UHSM, Hospital Universitario Jaén, Jaén; 2 USMC, Hospital de Antequera, Antequera; 3 USMC, Hospital Universitario Jaén, Jaén, Spain

## Abstract

**Introduction:**

Suicide is currently one of the biggest public health problems, it is the third cause of death in the age group between 15 and 29 years (16.36% of young people who died in 2013). The ‘Werther effect’ refers to the mimetic behavior of the suicidal act, thus making reference to the controversial novel “The Sorrows of Young Werther” by Goethe, in 1774. The population most susceptible to this influence is the most vulnerable and ambivalent, such as they can be adolescents and young people, people with personality disorders and drug use. Durkheim considered that imitation was not due to the contagion effect of making suicides public, but to the social conditions of some places, which were what caused people to commit suicide.

**Objectives:**

The objective of the case is to expose the vulnerability to the imitation of suicidal behaviors of young people suffering from personality disorder and drug use.

**Methods:**

We present the case of 4 young people between 18 and 21 years old (3 women and 1 man) from the same group of friends who, after the death by suicide of a 20-year-old boy, in the following 2 months, carried out suicidal behavior by taking medication they found at home and consumption of different drugs.

**Results:**

The two 21-year-old patients planned for the first month of the anniversary of the friend’s death, the intake of drugs and medication and leave a farewell note explaining the reasons. The patients required hospitalization in an acute mental health unit, one patient developed myocarditis secondary to toxins, during hospitalization they undergo psychotherapeutic treatment and are evaluated, leading to the diagnosis of Borderline Personality Disorder and Multiple Drug Use Disorder. The 20-year-old patient took medication on the anniversary month but did not require hospitalization. He underwent outpatient follow-up at a day hospital. During the therapeutic process, he was diagnosed with schizoid personality disorder. The 18-year-old patient required hospitalization for structured self-injurious ideation with a risk of acting out at 2 months, psychotherapeutic treatment was started and she was diagnosed with borderline personality disorder and harmful drug use. The 4 young people continue outpatient follow-up by both the community mental health unit and the addiction treatment center.

**Conclusions:**

We observe in the series of cases exposed, the vulnerability of young people suffering from personality disorders and drug use to suicidal behavior, so risk factors for their prevention must be identified and continue working on adequate information of suicidal acts, whether completed or not, to avoid imitation phenomena. In all cases, suicide should not be seen as a desirable alternative and strategies to cope with difficulties and emotional management should be offered and promoted, especially in this young population that is still developing and is more vulnerable.

**Disclosure of Interest:**

None Declared

